# Supplementary material for: A Critical View of the Application of the APEX Software (Aqueous Photochemistry of Environmentally-Occurring Xenobiotics) to Predict Photoreaction Kinetics in Surface Freshwaters
Source: Molecules. 2019 Dec 18;25(1):9. doi: 10.3390/molecules25010009 (PMC7017383; doi:10.3390/molecules25010009)
Supplement: Supplementary file 1 [file molecules-25-00009-s001.zip › 3.2.4_gcc-4.4.0.zip!/3.2.4_gcc-4.4.0/3.2.4_gcc-4.4.0/doc/octave/html/interpreter/Conventions.html]

Conventions - Untitled


Previous: Simple Examples,
Up: Introduction


---

### 1.3 Conventions

This section explains the notational conventions that are used in this
manual. You may want to skip this section and refer back to it later.

- Fonts- Evaluation Notation- Printing Notation- Error Messages- Format of Descriptions
